# A year in review: basic science

**DOI:** 10.1186/1546-0096-12-S1-I1

**Published:** 2014-09-17

**Authors:** Lucy R Wedderburn

**Affiliations:** 1 Infection, Immunity, Inflammation and Physiological Medicine Programme, UCL Institute of Child Health, London, UK

## 

The year of September 2013 - 2014 has seen some seminal discoveries in the field of human autoimmunity and immunology : work in Paediatric Rheumatology has led the way in several of these. In this session we will review together some of the exciting and important new developments in basic understanding of disease pathogenesis. We will also hear of the application of high throughput techniques and large datasets to our understandings of mechanism and to our translational goals towards improving lives of our patients.

However one small note of caution - it is never possible to review everything of high importance and relevance, so apologies in advance if some studies cannot be squeezed in

## Disclosure of interest

None declared.

